# The quality and reliability of TikTok videos on non-alcoholic fatty liver disease: a propensity score matching analysis

**DOI:** 10.3389/fpubh.2023.1231240

**Published:** 2023-10-04

**Authors:** Yongkang Lai, Zixuan He, Yilong Liu, Xiaojing Yin, Xuanming Fan, Ziang Rao, Hongyu Fu, Lun Gu, Tian Xia

**Affiliations:** ^1^Department of Gastroenterology, Changhai Hospital, Second Military Medical University/Naval Medical University, Shanghai, China; ^2^Department of Gastroenterology, Ganzhou People’s Hospital Affiliated to Nanchang University, Ganzhou, Jiangxi, China; ^3^College of Basic Medicine Sciences, Second Military Medical University/Naval Medical University, Shanghai, China

**Keywords:** nonalcoholic fatty liver disease, lifestyle modification, TikTok, health education, social media

## Abstract

**Background:**

Lifestyle modification is the cornerstone of non-alcoholic fatty liver disease (NAFLD) prevention and treatment. Short video platforms can facilitate easier access to health information for patients, thereby influencing lifestyle changes. An increasing number of individuals rely on online platforms to acquire health-related information about NAFLD. However, the quality of information regarding NAFLD on these platforms remains unclear.

**Objective:**

This study aimed to investigate the quality of information about NAFLD on TikTok.

**Methods:**

A total of 497 videos were retrieved from TikTok. The basic video information, including the video source, was extracted. Two independent raters evaluated the quality and reliability of the videos using the Global Quality Score system and a modified DISCERN tool. Propensity score matching (PSM) was used to compare video quality across sources.

**Results:**

NAFLD-related videos on TikTok were divided into three groups according to the uploader: health professionals, medical institutions, and science bloggers. Overall, the quality of NAFLD videos on TikTok was not satisfactory. Before PSM, there were no significant differences in video quality or content between the three groups. After PSM, the quality of NAFLD videos from health professionals was significantly better than the videos created by other groups. Besides, the videos of health professionals outperformed those of medical institutions and science bloggers in terms of the definition of disease, risk factors, and treatment, but were inferior to those of medical institutions considering the symptoms and tests of NAFLD.

**Conclusion:**

The quality of NAFLD-related videos on TikTok needs improvement. Compared with videos created by science bloggers and medical institutions, videos from health professionals may provide accurate guidance on the treatment and prevention of NAFLD.

## Introduction

Non-alcoholic fatty liver disease (NAFLD) is the most common chronic liver disease, with a global prevalence of 25%, and is the second leading indication for liver transplantation worldwide (1, 2). The disease ranges from steatosis to non-alcoholic steatohepatitis and is closely related to cirrhosis and liver-related complications including hepatocellular carcinoma and liver failure (3). Despite the prevalence of NAFLD and the proportion of patients with advanced liver disease expected to increase in the future (4), there is currently no licensed pharmacotherapy available for this disease. Lifestyle modification, including dietary management and aerobic exercise, is the cornerstone of NAFLD prevention and treatment (3). Ma et al. performed a prospective study and indicated that increased diet quality was associated with less liver fat accumulation and reduced risk for new-onset fatty liver (5). A prospective study of 293 patients found that a greater extent of weight loss induced by lifestyle changes could effectively mitigate the fatty degeneration of the liver, preventing NAFLD (6). Moreover, a recent meta-analyses that included 22 randomized controlled trials pointed out that lifestyle modification was significantly associated with improvements in biomarkers of NAFLD and can reduce hepatic steatosis (7). However, incorrect lifestyle adjustments not only fail to effectively ameliorate the condition but also undermine patients’ determination to manage the disease (8). A common complaint among non-alcoholic fatty liver disease patients is the lack of guidance on how to make lifestyle changes (8). Therefore, in order to achieve better treatment outcomes for non-alcoholic fatty liver disease patients, appropriate health education must be conducted in clinical practice to assist individuals in enhancing overall management.

In the digital age, the internet and social media have become important sources of healthcare information, and the emerging internet technologies providing people with health education are increasing in popularity (9, 10). Short video platforms, such as YouTube and TikTok, are one of the most popular types of social media nowadays. Compared with traditional textual information available in newspapers and books, these readily accessible platforms provide content in the form of graphic videos, allowing their users to absorb and remember the information more easily (11, 12). Moreover, health messages in the form of images can elicit an emotional response from consumers and motivate them to become healthy (13). Thus, providing information about NAFLD through social media platforms could be a promising approach to improving the prognosis of patients with NAFLD.

Recently, a study revealed that the number of people accessing information about NAFLD via the Internet was increasing every year, and the majority of patients considered Internet information to be trustworthy (14). However, many medical science videos come from lay users without medical professional training, which results in mixed information, and inaccurate or biased information may mislead patients (15, 16). Moreover, assessing the quality of online health information about NAFLD is not an easy task for most patients. Therefore, it is imperative for healthcare providers to assess the quality of online information about NAFLD and guide patients. TikTok is one of the most popular social media platforms, with nearly 800 million users and billions of views daily (17), and it is an important source of health information about NAFLD. However, the quality of NAFLD information on TikTok has yet to be sufficiently evaluated. To bridge this gap in research, this study aimed to investigate the information quality of videos on NAFLD on TikTok.

## Methods

### Search strategy and data processing

**Figure 1:**
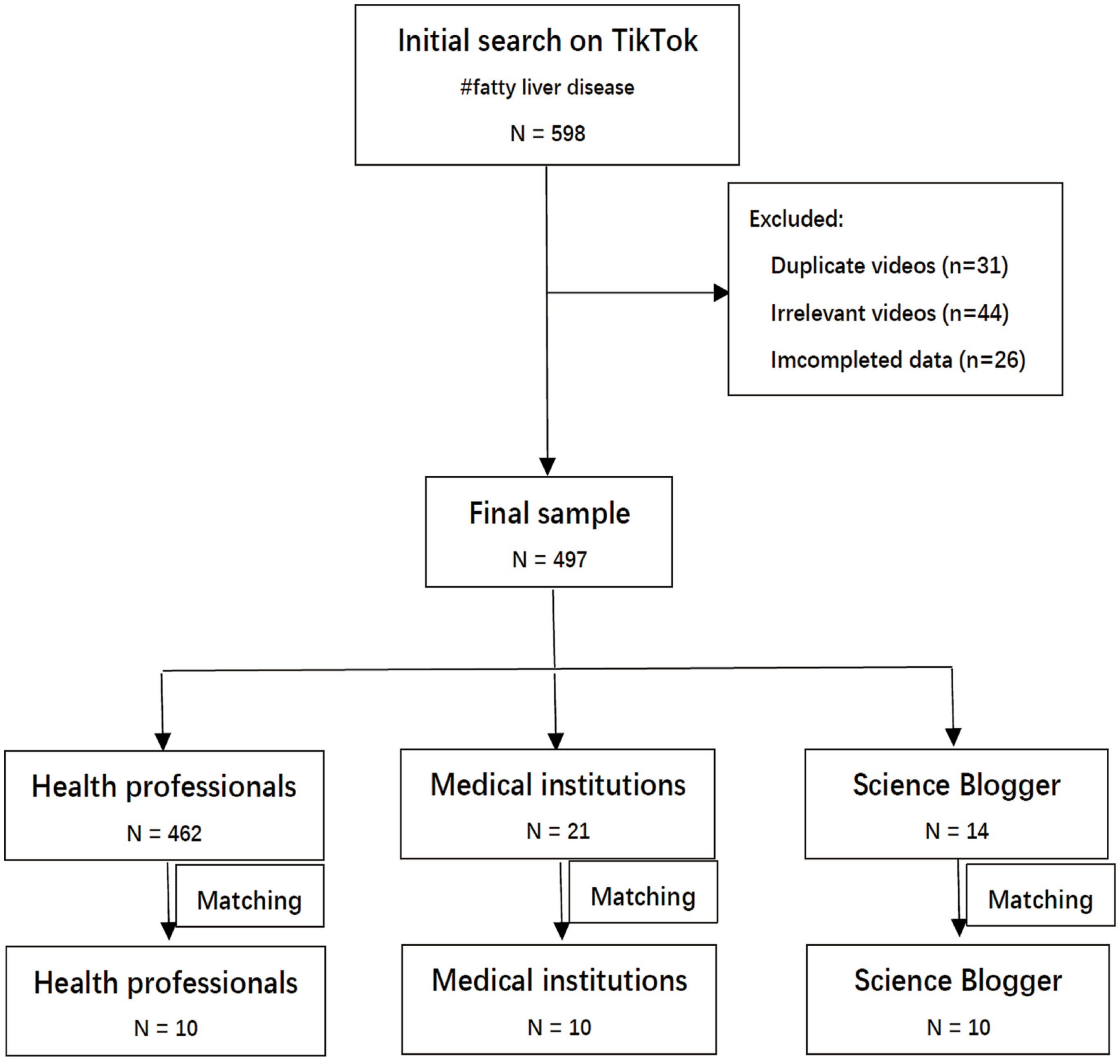
Flowchart of videos included in the present study.

This study was designed as an observational retrospective study of TikTok videos obtained from a smartphone. Relevant videos were gathered from TikTok (the Chinese version) during the period from December 2018 to September 2022. The search keyword was “非酒精性脂肪肝” (“nonalcoholic fatty liver disease” in Chinese). The exclusion criteria were as follows: duplicate videos, videos irrelevant to the topic, videos with no sound/poor sound quality, and videos with incomplete data were excluded from the study. Videos for commercial purposes and not in the Chinese language were also excluded. Two experienced gastroenterologists (HF and TX) reviewed and classified the videos independently, and any disagreement was resolved by consensus. Finally, 497 videos were included for data analysis (Figure 1).

Basic information was extracted from each video, including publication date, type of uploader, video duration (s), number of likes, number of favorites, and number of shares. All the data were recorded in Excel (Microsoft Corporation).

### Evaluation methodology and procedure

The content of each video was evaluated using the modified DISCERN scoring system and a coding schema (11). DISCERN is a validated instrument widely applied in research to help consumers and care providers evaluate the quality of health information (11, 13, 18). As shown in Supplementary Table S1, DISCERN evaluates the quality of video content through five questions; each question receives 1 point for a “yes” answer and 0 points for a “no” answer. The coding schema proposed rates the quality of six types of content: the definition of a disease, signs and symptoms, risk factors, evaluation, management, and outcomes. The videos were rated for each type of content on a three-item scale: no content (0 points), some content (1 point), and extensive content (2 points). Besides, we employed the Global Quality Score (GQS) to assess the reliability and quality of videos. The GQS is widely used for evaluating the quality of health information on online video platforms, and the videos are scored on a 5-point scale ranging from 1 (poor quality) to 5 (excellent quality) (13, 19, 20). The details of this instrument are given in Supplementary Table S2.

None of the videos were downloaded, retweeted, liked, or commented on before performing the search. We deleted all history and settings on the smartphone to avoid potential prebuffered cache-induced directional information recommendations. Two qualified physicians (Lai YK and He ZX) working in the department of gastroenterology in a tertiary hospital independently evaluated each video according to the following two stages. First, they recorded basic video information including publication date, video duration, number of likes, number of favorites, number of shares, and publisher information, which included account name, self-description, and identity verification status. The type of uploader was categorized into three main types (health professionals, medical institutions, and science bloggers) and identified by their account name and verification status. Second, they evaluated the content, reliability, and quality of the videos using DISCERN, the coding schema proposed, and the GQS. Before evaluating the videos, the rater reviewed the official instructions for the abovementioned instruments, discussed the best way to use these tools to evaluate the content of the videos, and made the necessary adjustments.

### Statistical analysis

Cohen’s coefficient (κ) was used to assess the overall rating agreement, which was performed according to a previous study (21). Cohen’s κ for this study was greater than 0.8, indicating good interrater reliability. All continuous variables in this study were abnormally distributed and, therefore, expressed as the median and interquartile range (IQR) and analyzed using the Mann–Whitney rank sum test when two medians were compared. Categorical variables were presented as proportions, and the chi-square test or Fisher’s exact test was used as appropriate. DISCERN, the six content categories from Goobie et al. (22) and the GQS were recorded as rank variables and compared using the Wilcoxon rank sum test.

Additionally, to control and reduce selection bias and confounders in this retrospective study, we performed a multi-group propensity score (PS) analysis as a non-randomized sensitivity analysis (23). The PS was estimated based on the following covariates in a multivariable logistic regression model: video duration, number of likes, number of favorites, and number of shares. The health professionals group was matched to the medical institutions group and the science bloggers group in a 1:1:1 ratio, utilizing the nearest neighbor method with a caliper width of 0.2. After matching, all baseline characteristics were balanced (*p* > 0.05) among the three groups. All statistical analyses were performed using the R statistical software 4.2.2,^1^ and a *p*-value of <0.05 was considered to be statistically significant.

## Results

### Video characteristics

According to the primary identity of the uploaders, we divided them into three groups: health professionals, medical institutions, and science bloggers. As shown in Table 1, health professionals contributed the greatest number of videos (*n* = 462, 93%), followed by medical institutions (*n* = 21, 4%) and science bloggers (*n* = 14, 3%). The duration of the videos varied from 5 to 438 s, and the median duration was 88 s (IQR: 66–122 s). While the median number of “likes,” “favorites,” and “shares” were 406 (IQR: 139–1,666), 32 (10–141), and 100 (24–476), respectively.

**Table 1:** Characteristics of the sources of fatty liver disease-related TikTok videos (*N* = 497).

Source	Source description	Videos, *n* (%)
Health professionals	Individuals who presented themselves as health professionals (included doctors and nurses)	462 (93)
Medical institutions	Health institutions established in accordance with legal procedures to engage in disease diagnosis and treatment activities	21 (4)
Science blogger	Individuals who presented themselves as health professionals and engaged in spreading scientific knowledge	14 (3)

### Video content

**Figure 2:**
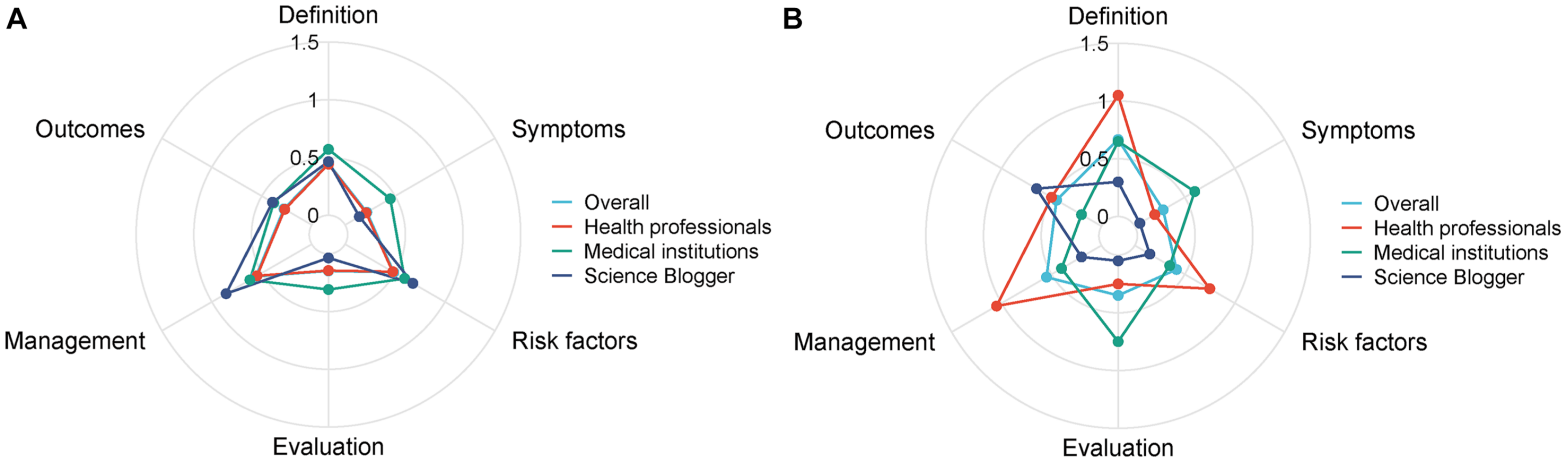
Comparisons of content comprehensiveness between different sources before **(A)** and after matching **(B)**.

We evaluated the information quality of the videos based on six types of content: definition of disease, signs/symptoms, risk factors, evaluation, management, and outcomes. The results showed that most of the videos contained no or little content about the disease. As shown in Table 2; Figure 2A, 91.5% of the videos contained no or little content on the signs/symptoms of NAFLD, and 76.4% of the videos did not specify (contained no or little content) the definition of NAFLD. Additionally, 71.4% of the videos failed to mention the risk factors for NAFLD. Notably, in our sample, there were no videos with exhaustive information (most content or extensive content) on the tests related to NAFLD and their outcomes, accounting for merely 0.2 and 1% of the videos, respectively. Overall, most of the videos are more focused on the management of NAFLD, accounting for 35.8% of the videos (some content, most content, or extensive content).

**Table 2:** Completeness of video content.

Video content	Definition, *n* (%)	Signs/symptoms, *n* (%)	Risk factors, *n* (%)	Test, *n* (%)	Treatment/management, *n* (%)	Outcomes, *n* (%)
No content (0 points)	191 (37.8)	332 (66.8)	176 (35.4)	386 (77.7)	157 (31.6)	282 (56.7)
Less content (0.5 points)	188 (38.6)	123 (24.7)	179 (36)	76 (15.3)	162 (32.6)	158 (31.8)
Some content (1 point)	99 (19.9)	35 (7)	124 (24.9)	34 (6.8)	150 (30.2)	52 (10.5)
Most content (1.5 points)	16 (3.2)	4 (0.8)	14 (2.8)	0	18 (3.6)	5 (1)
Extensive content (2 points)	3 (0.6)	3 (0.6)	4 (0.8)	1 (0.2)	10 (2)	0

### Information quality and reliability

As shown in Table 3, according to the DISCERN score, the general quality of the videos was not acceptable, with a median score of 2 (IQR: 2–3). We also evaluated the videos using the GQS system. However, consistent with the results of DISCERN, the reliability of the videos was also low, with a median GQS value of 2 (IQR: 2–3).

**Table 3:** Characteristics of fatty liver disease-related TikTok videos.

Characteristics	*n* = 497
Video duration (s), median, IQR	88 (66–122)
Number of likes, median, IQR	406 (139–1,666)
Number of favorites, median, IQR	32 (10–141)
Number of shares, median, IQR	100 (24–476)
DISCERN score, median, IQR	2 (2–3)
GQS score, median, IQR	2 (2–3)

### Comparison among health professionals, medical institutions, and science bloggers

Furthermore, we compared the content and quality of videos in the three subgroups of health professionals, medical institutions, and science bloggers using propensity score matching (PSM). Before PSM, as shown in Table 4, the science bloggers group posted videos with a longer duration (median: 96 s, IQR: 40–292 s) and more “shares” (median: 124, IQR: 21–619). However, the videos posted by the science bloggers group received fewer likes (median: 111, IQR: 10–777) than those posted by health professionals (median: 423, IQR: 152–1,760) and medical institutions (median: 256, IQR: 55–679), with *p* = 0.033. As for the quality of videos, medical institutions had higher DISCERN scores compared with the other two groups (*p* = 0.106). Regarding the video content, as shown in Figure 2A; Table 4, there was no significant difference among the three groups considering the definition of disease, signs/symptoms, risk factors, tests, treatment/management, and outcomes.

**Table 4:** Characteristics of fatty liver disease-related TikTok videos, by source.

Characteristics	Before matching				After matching			
	Health professionals (*n* = 462)	Medical institutions (*n* = 21)	Science blogger (*n* = 14)	*p*-value	Health professionals (*n* = 10)	Medical institutions (*n* = 10)	Science blogger (*n* = 10)	*p*-value
Video duration (s), median, IQR	88 (66–122)	88 (68–146)	96 (40–292)	0.543	89 (74–128)	83 (62–114)	92 (40–124)	0.67
Number of likes, median, IQR	423 (152–1,760)	256 (55–679)	111 (10–777)	0.033	193 (51–607)	263.5 (59–679)	73.5 (10–306)	0.301
Number of favorites, median, IQR	32 (10–140)	11 (8–130)	32 (7–317)	0.703	124 (43–736)	105 (15–323)	134 (21–944)	1
Number of shares, median, IQR	100 (24–481)	72 (29–242)	124 (21–619)	0.977	13 (5–50)	10 (3–130)	110 (10–317)	0.392
GQS score^a^	245.66	300.21	282.36	0.093	22.35	13.2	10.95	0.004
DISCERN score^a^	244.42	331.31	276.64	0.106	20.65	14.85	11	0.033
Definition^a^	247.37	288.76	243.25	0.383	21.7*	15.4*	9.4*	0.005
Signs/symptoms^a^	247.88	292.36	220.89	0.164	14.7	20.05*	11.75*	0.002
Risk factors^a^	246.29	279.57	292.57	0.26	21.2*	14.5*	10.8*	0.016
Test^a^	248.31	290.19	210	0.052	14.5	20.8*	11.2*	0.016
Treatment/management^a^	245.74	260.69	339.14	0.14	22.9*	13.5*	10.1*	0.002
Outcomes^a^	247.52	263.5	276.04	0.616	16.85	11.65	18	0.182

a These variables were recorded as rank variables and comparisons were made by Wilcoxon’s rank-sum test.

*Statistically significant comparison.

**Figure 3:**
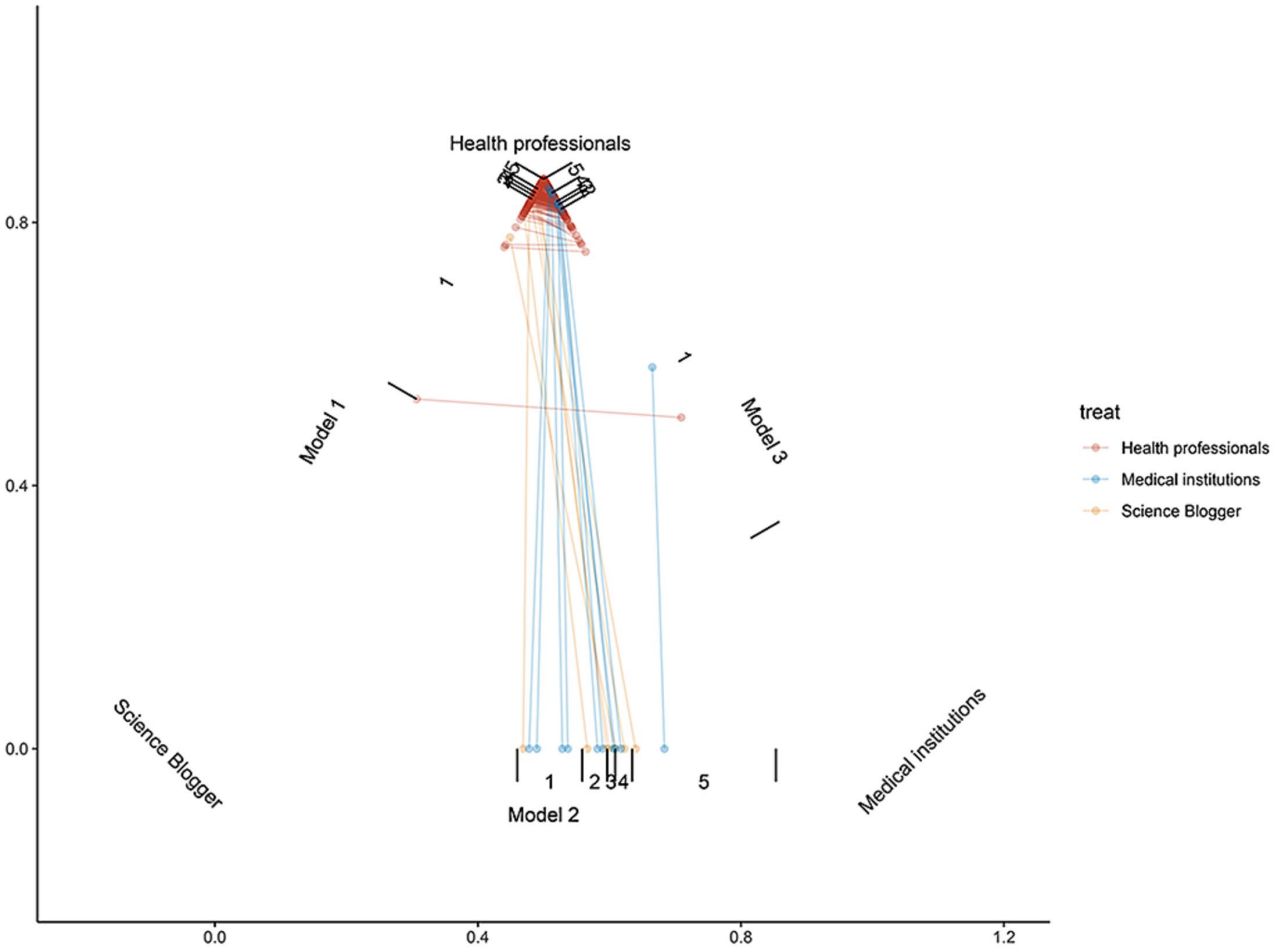
Multi-group propensity matching ternary graph, which visually examines the overlapping assumptions of the data. The graph represents the range of propensity matching values between the groups of healthy individuals, medical institutions, and science bloggers without equal parts.

After PSM, 10 videos were assigned to each of the three groups, and the bias of each group was reduced (Figure 3). There were no significant differences among health professionals, medical institutions, and science blogger groups in terms of video duration, number of likes, number of favorites, and number of shares. As shown in Table 4, according to the GQS system and DISCERN score, the average rank of the quality of videos provided by health professionals (GQS: 22.35; DISCERN: 20.65) was significantly higher than those provided by medical institutions (GQS: 13.2; DISCERN: 14.85) and science bloggers (GQS: 10.95; DISCERN: 11) (GQS *p* = 0.004; DISCERN *p* = 0.033). Besides, the content on the definition of disease, risk factors, and management was more comprehensive in the videos provided by health professionals (average rank, definition of disease: 21.7; risk factors: 21.1; management: 22.9) than in the videos provided by medical institutions (average rank, definition of disease: 15.4; risk factors: 14.5; management: 13.5) and science bloggers (average rank, definition of disease: 9.4; risk factors: 10.8; management: 10.1) (definition of disease *p* = 0.005; risk factors *p* = 0.016; management *p* = 0.002) (Table 4; Figure 2B). However, in terms of signs/symptoms and tests, the videos provided by medical institutions (average rank, signs/symptoms: 20.05; tests: 20.8) were significantly more detailed than the videos provided by health professionals (average rank, signs/symptoms: 14.7; tests: 14.5) and science bloggers (average rank, signs/symptoms: 11.75; tests: 11.2) (signs/symptoms *p* = 0.002; tests *p* = 0.016).

## Discussion

### NAFLD can be prevented and even cured through lifestyle changes

NAFLD is a clinicopathological syndrome of predominantly hepatic steatosis, excluding alcohol and other definite liver damage factors, which includes simple fatty liver, as well as its evolution into steatohepatitis, fatty liver fibrosis, and even cirrhosis and hepatocellular carcinoma (24). NAFLD is becoming a major health challenge, with the worldwide prevalence ranging from 28.1 to 52.34 per 1,000 person-years (25, 26). NAFLD is mainly associated with a high-energy diet and a lack of exercise. Men and those suffering from obesity, type 2 diabetes, and hyperlipidemia are more susceptible to developing NAFLD (27). According to the recent consensus recommendations from the British Association for the Study of the Liver, weight reduction through regular exercise and caloric restriction is fundamental in managing NAFLD (3). Numerous studies have shown that weight loss up to 5% can improve steatosis; weight loss up to 7–10% can positively affect NAFLD activity scores and liver fibrosis; and weight loss greater than 10% can significantly improve steatohepatitis as well as liver fibrosis (6, 28–30). Therefore, reducing the intake of high-calorie foods and increasing exercise frequency, especially for people at higher risk of NAFLD, can be effective in preventing the development of NAFLD. Through the right lifestyle modifications, it is possible to treat simple fatty liver and reverse steatohepatitis (31, 32).

### High-quality health education can help patients with NAFLD improve their lifestyle

Health education is important in maintaining public health levels, and its significance has increased especially during the COVID-19 pandemic. High-quality health education not only helps patients to fully understand the disease so that they can seek early interventions for prevention or self-care and work with medical professionals to become active participants in their own treatment, but also minimizes complications, reduces the burden of healthcare costs, and allows for the rational and effective use of health resources, particularly for NAFLD, a disease that can be effectively prevented and treated through lifestyle changes (11, 33, 34). However, there is still a lack of public awareness about the potential severity of NAFLD, and abundant evidence shows that interventions aimed at modifying lifestyle-related risk factors are central to the treatment of NAFLD and that these interventions can prevent disease progression and, in some cases, reverse fibrosis (34–38). In particular, effective care of patients with diabetes mellitus combined with NAFLD, effective dietary improvement, and achieving and maintaining at least 10% weight loss will undoubtedly significantly improve the prognosis of patients with NAFLD and have important implications for the maintenance of world health and the rational use of global healthcare resources (33).

### Principal findings

As technology is gradually penetrating and influencing almost every aspect of our lives, health education and health promotion are also becoming digital (39). Compared to text-based information, video is becoming an important source of information for patients by presenting complex health information in a more understandable and impressive visual format (20, 40). As the largest short video platform in China, TikTok is playing an important role in the health education of people (19, 41).

In this study, we evaluated the content, quality, and reliability of health education videos about NAFLD on TikTok. The results showed that the overall quality of health education videos about NAFLD on TikTok was not satisfactory, which may be related to the fact that the content was not effectively supervised before it was released. Based on the identity of the video publishers, we classified the videos about NAFLD on TikTok into three main sources: health professionals, medical institutions, and science bloggers. Though the majority of videos were posted by health professionals, the content quality was still poor. First, the definition and description of NAFLD were incomplete and not accurate. Second, although the videos mainly focused on the risk factors and treatment methods of NAFLD, the content contained too much medical jargon, which is difficult for patients to understand, and the descriptions of the risk factors and how to treat and prevent NAFLD were also not clear. For example, most of the videos only told patients that they should exercise more but did not specify the frequency or level of exercise. Third, there were few videos on tests and the prognosis of NAFLD. This may be because the creators of these videos, although defined as professionals, were mostly physicians practicing emergency medicine, Chinese medicine, and cardiology, who were less knowledgeable about NAFLD than digestive health professionals.

In addition, we also compared the video content from different sources using PSM analysis and found that the overall quality of videos created by medical professionals was better than those developed by medical institutions and science bloggers. The videos created by medical professionals outperformed those created by medical institutions and science bloggers in terms of the definition of disease, risk factors, and treatment, but were inferior to those of medical institutions regarding symptoms and tests. This may be because medical professionals mainly focus on informing patients about the prevention and treatment of NAFLD, while medical institutions, which are for-profit, are more focused on informing patients about testing for NAFLD. The science bloggers group created the lowest-quality videos. These findings indicate that the video content of NAFLD on TikTok needs to be improved. We call on government departments and public hospitals to improve health education about NAFLD and ask doctors specializing in digestive health to provide better-quality videos on how to prevent and treat NAFLD. Besides, people should exercise caution when using TikTok to acquire information about NAFLD.

## Limitations and future directions

There are some limitations to this study. First, this study only included Chinese videos on TikTok; therefore, the findings of this study may not be applicable to videos from other platforms. Second, this study lacked information related to the departments and titles of health professionals; therefore, we could not analyze whether the quality of videos posted by physicians specializing in gastroenterology was significantly higher than those posted by non-gastroenterologists. However, this is the first study to evaluate the quality of NAFLD-related health education videos on TikTok and can guide future research in this field. The findings of this study can help patients who want to find information about NAFLD on TikTok to find videos with higher quality. Meanwhile, patients can also be guided to make the right lifestyle changes, which can reduce medical costs and reduce the burden of the disease in the country. It is hoped that more studies with large samples and in multiple languages will be conducted in the future to further consolidate our findings and provide directions for the future development of health education-related short videos.

Although the development of online information technology has made it possible for people to access complex health education information in a simple way by watching short videos, there are serious concerns about the quality of the videos. In the future, to improve the quality of health education videos and enable patients to receive effective and high-quality health education, the following steps should be taken: first, the government and online video platforms should establish a supervision and screening organization composed of health professionals, and videos should be screened before uploading. Notably, recent studies have confirmed that machine learning can play an important role in recognizing video quality (42–44). Therefore, in the future, the development of a plug-in that can only recognize the quality of user-uploaded videos by machine learning method seems to be an effective way to achieve video quality regulation. Second, the government and video platforms should encourage expert professionals to produce health education videos. If possible, the government or relevant institutions should invite experts to record authoritative videos, which should then be officially published on a unified channel to provide people with the right guidance. Finally, medical professionals should try to avoid using specialized vocabulary that is difficult to understand in the videos.

## Conclusion

The overall quality of health education videos about NAFLD on TikTok is unsatisfactory. The videos created by healthcare professionals have a higher quality than those created by medical institutions and science bloggers. In terms of the risk factors and treatment of NAFLD, the videos created by healthcare professionals are more detailed, while the videos from medical institutions have detailed information regarding how to test for NAFLD. Short videos have become an important source of health education, and it is imperative to formulate rules for publishing short health education videos on relevant online platforms to maintain public health.

## Data availability statement

The raw data supporting the conclusions of this article will be made available by the authors, without undue reservation.

## Author contributions

TX, LG, and HF: concept and design. YKL, ZH, YLL, and XY: data collection and analysis, with extensive contributions from ZR. YKL and ZH: drafting of the article. TX: critical revision of the article for important intellectual content. TX, LG, and HF: study supervision. XF: revising the article. All authors contributed to the article and approved the submitted version.

## Footnotes

^1^www.r-project.org

## Abbreviations

NAFLD: Non-alcoholic fatty liver disease
PSM: Propensity score matching
PS: Propensity score
IQR: Interquartile range
GQS: Global Quality Score
