# Supplementary material for: The quality and reliability of TikTok videos on non-alcoholic fatty liver disease: a propensity score matching analysis
Source: Front Public Health. 2023 Oct 4;11:1231240. doi: 10.3389/fpubh.2023.1231240 (PMC10582932; doi:10.3389/fpubh.2023.1231240)
Supplement: Supplementary file 1 [file Table_1.docx]

**Supplementary Table 1: Modified quality standards of discrimination (scored by answering the following questions, 1 point for "yes", 0 points for "no")**

| **Reliability Score** |
| --- |
| 1. Is the video clear, concise and easy to understand? |
| 2. Is the content presented balanced and unbiased? |
| 3. Does the video have a valid citation? |
| 4. Are there other sources of content listed? |
| 5. Whether the unknown areas of the direction are not mentioned? |
